# MSX2 in ameloblast cell fate and activity

**DOI:** 10.3389/fphys.2014.00510

**Published:** 2015-01-05

**Authors:** Sylvie Babajko, Muriel de La Dure-Molla, Katia Jedeon, Ariane Berdal

**Affiliations:** ^1^Laboratory of Molecular Oral Pathophysiology, Centre de Recherche des Cordeliers, Institut National de la Santé et de la Recherche Médicale, UMRS 1138Paris, France; ^2^Laboratory of Molecular Oral Pathophysiology, Centre de Recherche des Cordeliers, Université Paris-DescartesParis, France; ^3^Laboratory of Molecular Oral Pathophysiology, Centre de Recherche des Cordeliers, Université Pierre et Marie Curie-ParisParis, France; ^4^Laboratory of Molecular Oral Pathophysiology, Centre de Recherche des Cordeliers, Université Paris-DiderotParis, France; ^5^Centre de Référence des Maladies Rares de la Face et de la Cavité Buccale MAFACE, Hôpital RothschildParis, France

**Keywords:** MSX2, transcription factors, ameloblast, differentiation, enamel, teeth

## Abstract

While many effectors have been identified in enamel matrix and cells via genetic studies, physiological networks underlying their expression levels and thus the natural spectrum of enamel thickness and degree of mineralization are now just emerging. Several transcription factors are candidates for enamel gene expression regulation and thus the control of enamel quality. Some of these factors, such as MSX2, are mainly confined to the dental epithelium. MSX2 homeoprotein controls several stages of the ameloblast life cycle. This chapter introduces MSX2 and its target genes in the ameloblast and provides an overview of knowledge regarding its effects *in vivo* in transgenic mouse models. Currently available *in vitro* data on the role of MSX2 as a transcription factor and its links to other players in ameloblast gene regulation are considered. MSX2 modulations are relevant to the interplay between developmental, hormonal and environmental pathways and *in vivo* investigations, notably in the rodent incisor, have provided insight into dental physiology. Indeed, *in vivo* models are particularly promising for investigating enamel formation and MSX2 function in ameloblast cell fate. MSX2 may be central to the temporal-spatial restriction of enamel protein production by the dental epithelium and thus regulation of enamel quality (thickness and mineralization level) under physiological and pathological conditions. Studies on MSX2 show that amelogenesis is not an isolated process but is part of the more general physiology of coordinated dental-bone complex growth.

## Structure and molecular mechanisms of muscle segment homeobox (Msx) genes

### Homeobox genes

*Msx2* is a member of the family of divergent homeobox-containing genes homologous to the Drosophila *M*uscle *S*egment *H*omeobox (*msh*) gene. Evolution including the duplication of the ancestral *msh* gene, has led to three different genes in mice and two in humans. Homeobox-containing genes share a well-conserved sequence of 183 bp coding for a helix-loop-helix motif of 64 amino acids (Shirasawa et al., 1994). This homeodomain interacts with an A/T-rich DNA sequence thereby conferring transcriptional activity on the proteins carrying it (Gehring et al., 1994). Most homeobox genes are organized in clusters, and this is the case for *HoxA*, *B*, *C*, and *D* genes that control the development of the trunk spatially and temporally. Other homeobox genes, dispersed around the genome and classified as divergent homeogenes also include the *Msx* family which is crucial for the development of the head.

### MSX1 and MSX2 are transcriptional regulators

The homeodomain of homeogenes *Msx1* and *Msx2* share 98% sequence identity, such that they have similar transcriptional properties (Catron et al., 1996). MSX1 and MSX2 were first reported as transcriptional repressors (Catron et al., 1993, 1995), but their respective activities have not been precisely characterized. They are able to interact with a C/GTAATTG core consensus sequence (Catron et al., 1993). MSX homeoproteins may form either homodimers or heterodimers with other homeoproteins such as those encoded by *Dlx* (Zhang et al., 1997) and *Pax* genes (Bendall et al., 1999; Ogawa et al., 2006). The resulting competition for the same promoter sequence may explain, at least in part, their antagonist regulatory activities (Bendall and Abate-Shen, 2000). In addition to the presumed direct interactions via the homeodomain, their N- and C-terminal domains are able to interact with other proteins and thereby also modulate transcription (Catron et al., 1995; Zhang et al., 1996). MSX2 is able to interact with SP3 (Bei, 2009) and with C/EBPα, notably in ameloblasts (Zhou and Snead, 2000). Such physical interactions between MSX2 and C/EBPα enable switching of cell differentiation, as described for osteogenic/adipogenic differentiation in aortic myofibroblasts (Cheng et al., 2003). Transcriptional repression by MSX (reported for MSX1) is also modulated by interactions that drive the nuclear localization of the proteins, as shown for PIAS-1 (Lee et al., 2006) and H3K27me3 (Wang and Abate-Shen, 2012). These various papers show that the target selectivity of MSX1 and MSX2 and their transcriptional activities are dependent on the nature of the partners they interact with, via binding motifs located outside the homeodomain (Catron et al., 1995; Zhang et al., 1996). The sequence similarity between MSX1 and MSX2 in the N- and C-termini is lower (than in their homeodomains) and this presumably explains the different activities of the two factors. Transcriptional regulations of MSX2 depend on the nature of its partners: the specific combinations involved determine when, where and how the expression of the various MSX2-target genes is modulated.

### MSX1 and 2 present redundant and non-redundant functions

MSX functions are significant in morphogenesis in which specific developmental patterns control distinct stages and events. The involvement of MSX in morphogenesis was discovered in work with limb buds and ectodermal appendages such as teeth (Satokata and Maas, 1994; Houzelstein et al., 1997; Satokata et al., 2000). There are now a vast number of human and transgenic mouse gene mutations available (Table 1) providing evidence that both MSX1 and MSX2 are essential for skeletal morphogenesis and differentiation. The expression profiles of *Msx* homeobox genes may overlap; there may be redundancy between MSX1 and MSX2 as they display structural conservation, according to anatomical site (Sharpe, 1995). This is the case in limbs (and the resulting appendicular skeleton) but not in craniofacial morphogenesis and differentiation as illustrated by the selective phenotype of *Msx* mutants.

**Table 1:** ***MSX* mutations in human and corresponding experimental models**.

	**Human**		**Mutant mice**	**References**
	**Pathology**	**OMIM**	**Phenotype**	
MSX1	Ectodermal dysplasia 3, Witkop type	189500	*Loss of function*_(*Msx1*−/−)	
	Orofacial cleft 5	608874	Tooth agenesis, cleft palate	Satokata and Maas, 1994
	Tooth agenesis, with or without orofacial cleft	106600		Houzelstein et al., 1997
			*Gain of function* (transgenic mice) craniofacial bone morphogenesis	Nassif et al., 2014
MSX2	Loss of function	168550	*Loss of function* (*Msx2*−/−)	
	Parietal foramina with cleidocranial dysplasia		Bone defects	Satokata et al., 2000
			Tooth	Aïoub et al., 2007
				Molla et al., 2010
	Gain of function	604757	*Gain of function* (transgenic mice)	Liu et al., 1995
	Craniosynostosis, type 2		Premature suture closure,	Shao et al., 2005
			Ectopic cranial bone	
			Cardiovascular calcification	

Non-redundant roles of MSX are exemplified in teeth. Both MSX1 and MSX2 are expressed as soon as dental lamina is initiated and their expression continues until the end of tooth formation, but in different areas (Thesleff, 2003). Transgenic mouse models have been used to characterize their respective function in dental development: MSX1 drives early tooth morphogenesis, whereas MSX2 is involved later in development (Bei et al., 2004; Thesleff, 2006). *MSX1* gene mutations are associated with tooth agenesis in both human and mouse species (Vastardis et al., 1996; Houzelstein et al., 1997). *Msx2* null mutants survive and display variable bone and dental phenotypes, especially in areas in which development is driven by epithelial-mesenchymal cell communications (Satokata et al., 2000). The different phenotypes associated with either MSX1 or MSX2 transgenic models strongly indicate that they do not have the same functions in tooth development; their respective molecular actions and partners need to be characterized.

## MSX2: a key element of the transcriptional network determining ameloblast gene expression and ameloblast life-cycle

### MSX2, a key factor for enamel gene expression

**Figure 1:**
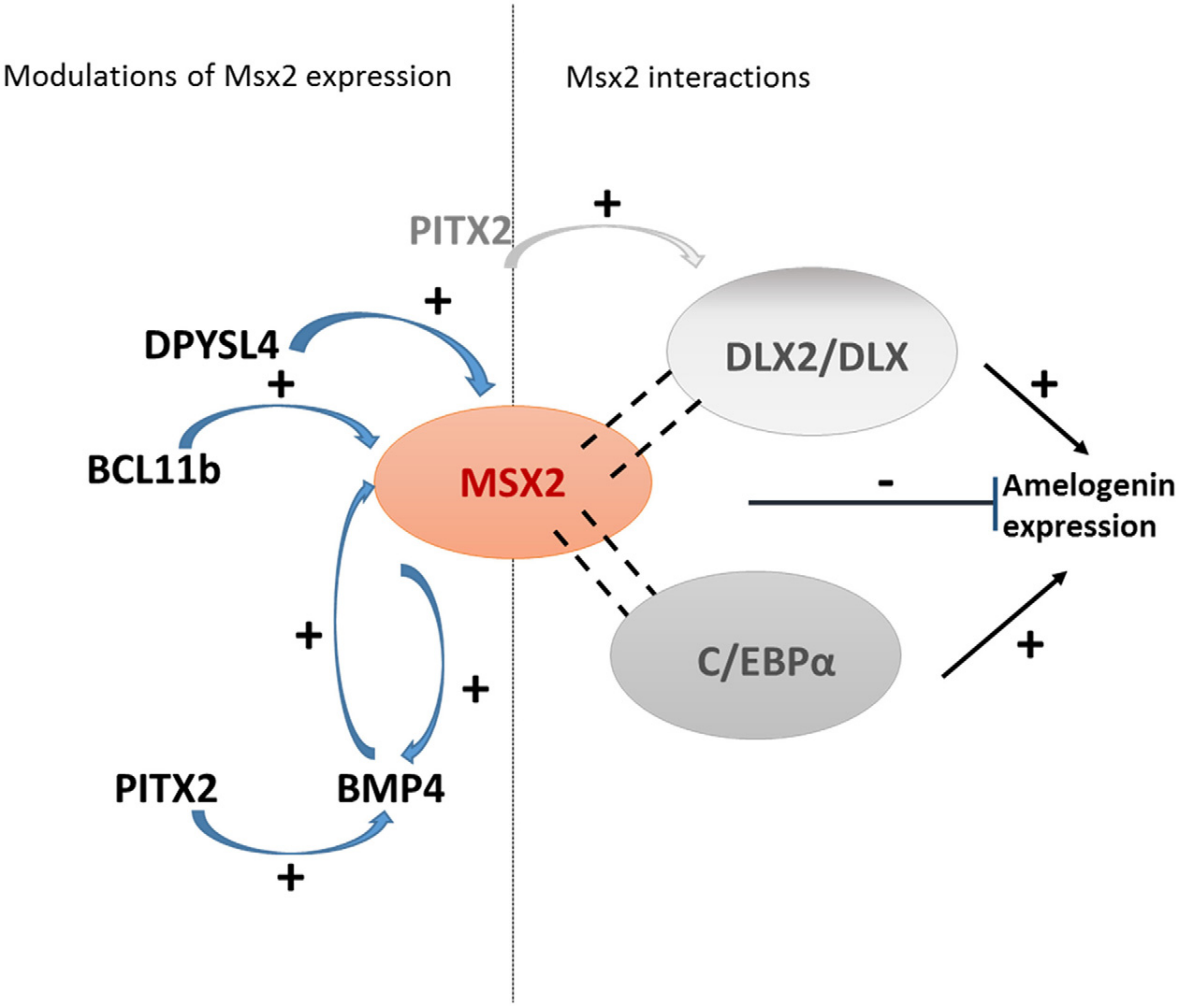
**The central role of MSX2 in ameloblast cell fate**. *Msx2* expression is controlled by transcription factors involved in ameloblast proliferation. MSX2 modulates the expression of its target genes directly or indirectly by interacting with various partners such as C/EBPα or Dlx (Zhou and Snead, 2000; Lézot et al., 2002, 2008). Thus, it represses the transcriptional activity of the transcription factors that modulate amelogenin expression.

A number of studies have investigated the regulation of enamel gene transcription. They have identified a number of factors, including MSX2, controlling the transcription of enamel genes (Table 2). The amelogenin gene was the first to be studied because it encodes the most abundant enamel matrix protein. Amelogenin gene repression by MSX2 appears to be indirect through interaction with C/EBPα (Figure 1) (Zhou et al., 2000; Xu et al., 2007a). Interaction between MSX2 and C/EBPα abolishes the activating activity of C/EBPα on amelogenin transcription. MSX2/DLX heterodimers may also be involved in modulating amelogenin expression (Lézot et al., 2008). Indeed, DLX2 and DLX3 have been shown to control amelogenin expression (Lézot et al., 2008; Athanassiou-Papaefthymiou et al., 2011). The selective role of each DLX homeoprotein may be influenced by the other DLX family members expressed in ameloblasts (Lézot et al., 2008). MSX2 transcriptional modulations have been documented for other key enamel genes, notably those encoding enamel matrix proteins (enamelin and ameloblastin) and the two main proteases (MMP20 and KLK4) (Berdal et al., 1993; Molla et al., 2010). MSX2 is also able to repress the expression of calbindin-D_28k_, a vitamin D-dependent calcium-handling protein, by direct interaction with its proximal promoter (Bolaños et al., 2012). Several partners of MSX2 identified in osteoblasts also influence ameloblast gene expression: for example, RUNX2 differentially regulates enamelin and *Klk4* gene expression. Interestingly, its own expression is up-regulated by NR1D1 which expression is also controlled by clock genes (Athanassiou-Papaefthymiou et al., 2011) establishing a complex network of direct and indirect controls by circadian transcription factors. Thus, clock proteins (BMAL1, Clock, PER1, and PER2) may regulate enamel gene expression either directly (Lacruz et al., 2012a; Zheng et al., 2013) or indirectly via NR1D1 and consequently MSX2. *ODAM* expression is also up-regulated by RUNX2, and ODAM increases *Mmp20* expression with its promoter activity (Lee et al., 2010). In view of the relationship between MSX2 and RUNX2, it may be possible that MSX2 also modulates *Mmp20* expression.

**Table 2:** **Transcriptional regulations of the major enamel genes**.

**Target genes**	**Transcription factors**	**References**
Amelogenin	Msx2 ↘	Zhou et al., 2000; Xu et al., 2007a
	C/EBPα ↗	Zhou and Snead, 2000
	NF-Y ↗	Xu et al., 2006
	C/EBPΔ ↗	Xu et al., 2007b
	Dlx2 ↗	Lézot et al., 2008
	Dlx2 and FoxJ1 ↗	Venugopalan et al., 2011
	Oct-1 ↘	Xu et al., 2010
	Pitx2 ↗	Li et al., 2013, 2014
	Clock genes ↗	Lacruz et al., 2012a; Zheng et al., 2013
	Runx2 ↘ and Dlx3 ↗	Athanassiou-Papaefthymiou et al., 2011
		Mitsiadis et al., 2008
	Tbx1 ↗	
Ameloblastin	Cbfa1 (Runx2) ↗	Dhamija and Krebsbach, 2001
Enamelin	B-catenin/LEF1 ↗	Tian et al., 2010
	Runx2 ↘ and Dlx3 ↗	Athanassiou-Papaefthymiou et al., 2011
MMP20	Runx2 and ODAM ↗	Lee et al., 2010
	c-Jun (AP1) ↗	Zhang et al., 2007
KLK4	Clock genes ↗	Zheng et al., 2013
	Runx2 ↗ and Dlx3 ↗	Athanassiou-Papaefthymiou et al., 2011
Calbindin D 28k	Msx2 ↘	Bolaños et al., 2012

**Figure 2:**
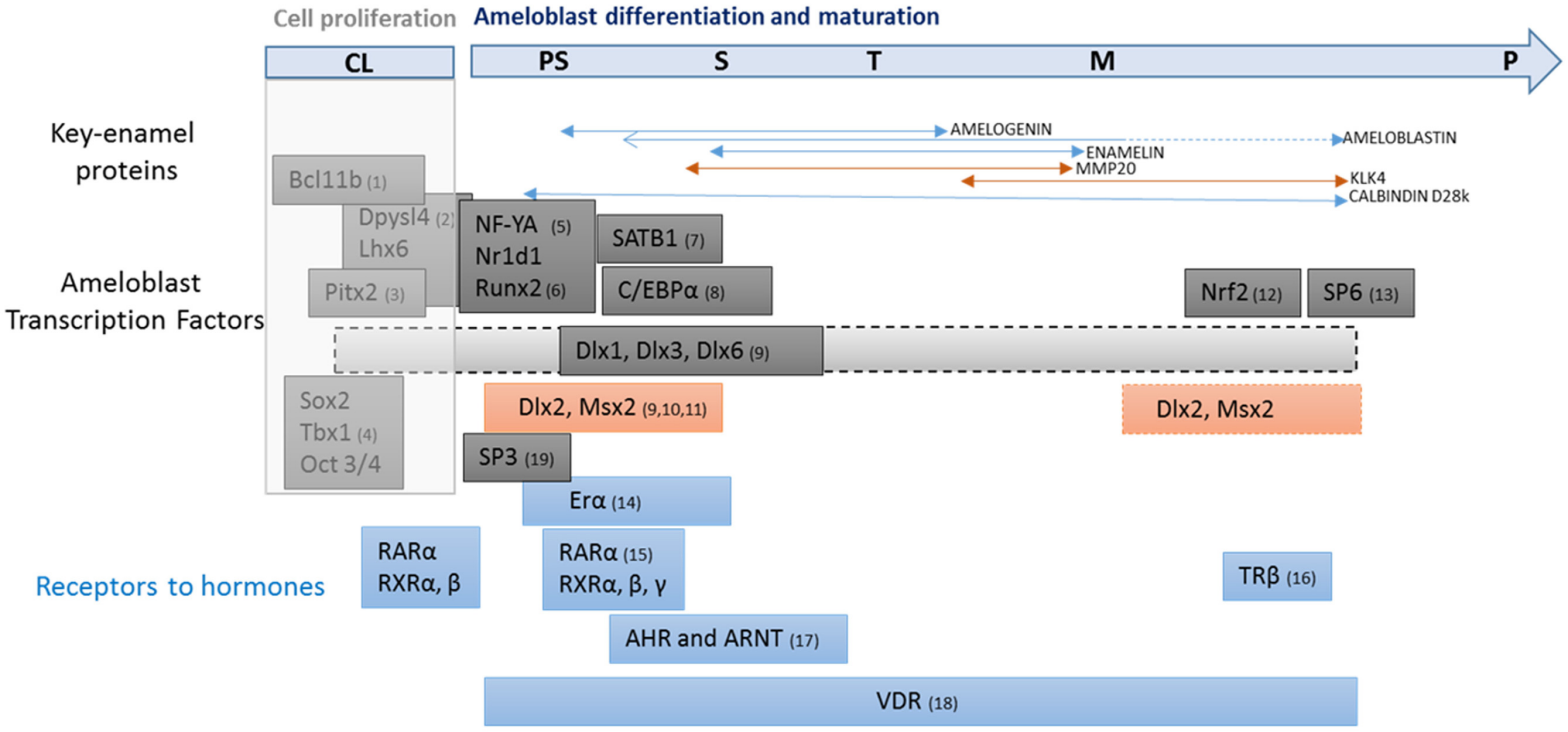
**Transcription factors involved in ameloblast proliferation, differentiation and maturation**. Transcription factors involved in ameloblast proliferation and differentiation (in black), and hormonal response (in blue). Enamel gene patterns are linked to presecretion, secretion and maturation stages of amelogenesis. The first key-point is the transition from presecretion to secretion stage during which differentiated ameloblasts acquire all the properties required for orderly secretion of enamel proteins (amelogenin, enamelin, amelobastin, and calbindin-D_28k_). A subsequent key-point for amelogenesis is the second transition from secretion to post-secretion. This event determines final enamel thickness via an abrupt decrease in matrix protein production. Enamel mineral quality is also conditioned by this transition associated with massive cell apoptosis and size reduction. Ameloblasts show an abrupt increase in the production of proteins involved in the mineralization process, including MMP20 and KLK4 proteases, mineral-handling effectors such as Ca-ATPase, alkaline phosphatase, proton pumps, carbonic anhydrase, calbindin-D_28k_, and tight junction elements which contribute to enamel maturation. The list of up- and down-regulated genes at these two key stages of amelogenesis is emerging from current “omics” studies and most of them have been identified. The challenge now will be to integrate these effectors and their regulators into a model that describes the resulting enamel quality. **CL**, cervical loop; **PS**, pre-secretion; **S**, secretion; **T**, transition; **M**, maturation stages and **P**, pigmentation. (1, Golonzhka et al., 2009; 2, Yasukawa et al., 2013; 3, Cao et al., 2013; 4, Catón et al., 2009; 5, Xu et al., 2007a; 6, Athanassiou-Papaefthymiou et al., 2011; 7, Stahl et al., 2013; 8, Zhou and Snead, 2000; 9, Lézot et al., 2008; 10, Bei et al., 2004; 11, Molla et al., 2010; 12, Yanagawa et al., 2004; 13, Muto et al., 2012; 14, Ferrer et al., 2005; 15, Bloch-Zupan et al., 1994; 16, Lacruz et al., 2012b; 17, Sahlberg et al., 2002; 18, Davideau et al., 1996; 19, Bei, 2009).

In addition, as evidenced in early development, *Msx2* expression itself may be controlled either directly or indirectly via enamel proteins. This was demonstrated by *in vivo* and *in vitro* studies showing a feedback loop between ameloblastin and *Msx2* expression (Fukumoto et al., 2004; Sonoda et al., 2009). However, the best characterized positive feedback loop involving MSX2 and a secreted protein is that operating with BMP4: BMP4 induces *Msx2* expression and in turn, MSX2 controls *Bmp4* (Bei and Maas, 1998) (Figure 2). Indeed, the *Msx2* promoter contains a BMP-responsive enhancer element (Brugger et al., 2004) and the *Bmp4* promoter contains an Msx (1 and 2) responsive element (Wang et al., 2011).

Considering the redundancies between *Msx1* and *Msx2*, it is interesting to raise the question of similar expression modulations for *Msx2* compared to those already reported for *Msx1*. Also, the level of Msx1 protein is regulated by its own antisense RNA: the *Msx1* gene is transcribed in both directions producing, in addition to the sense transcript, a long endogenous antisense non-coding RNA (Blin-Wakkach et al., 2001). This RNA is believed to provide fine control of MSX1 homeoprotein quantities during development (Coudert et al., 2005) via post-transcriptional sense RNA decay (Petit et al., 2009) and thus influence MSX1 protein activity (Babajko et al., 2009). Number of *Hox* homeogenes are submitted to bi-directional transcription (Mainguy et al., 2007); similar events that need to be investigated may also control *Msx2* expression.

In conclusion, *Msx2* expression is modulated either directly by various intracellular factors or indirectly by secreted factors, resulting in fined tuned levels of MSX2 that control enamel gene expression and ameloblast cell fate.

### Msx2 is involved in cell proliferation

MSX2 is present throughout the process of ameloblast differentiation/maturation although its expression decreases during the secretory stage and may modulate enamel gene expression differently depending on the combination of transcription factors present (Figure 2). Very few studies report gene expression modulations during the maturation stage (Lacruz et al., 2012b), such that the function of MSX2 during the terminal differentiation of ameloblasts is less clear.

*Msx2* is not only expressed in the inner dental epithelium but throughout the entire enamel organ as evidenced in the rodent apical loop (Jiang and Wang, 2010). In *Msx2*−/− mouse dental epithelium, proliferation of stellate reticulum cells and stratum intermedium cells increases. At these early stages, *Msx2* expression is induced by the key transcription factors, DPYSl4 and BCL11b (Ctip2), also determinant of cell proliferation (Figures 1, 2) (Golonzhka et al., 2009; Yasukawa et al., 2013). *Msx2* expression and *Dlx2* expression are also indirectly up-regulated by PITX2 via BMP4, which are also expressed in proliferative cells (Venugopalan et al., 2011; Cao et al., 2013) (Figure 1). As a result of the various *Msx2* expression modulations by early factors, *Msx2* is expressed in undifferentiated inner enamel epithelial (IEE) cells and down-regulated when ameloblast cells overtly differentiate (Mackenzie et al., 1991, 1992; Maas and Bei, 1997).

## Lessons from non-conditional *Msx2* mutants—strengths and limitations

Most of the data described above result from *in vitro* studies. However, *in vivo* models are also informative about MSX2 function because they include cell-cell communications.

### *Msx2*−/− mouse dental phenotype illustrates the pleiotropic role played by *Msx2*

**Figure 3:**
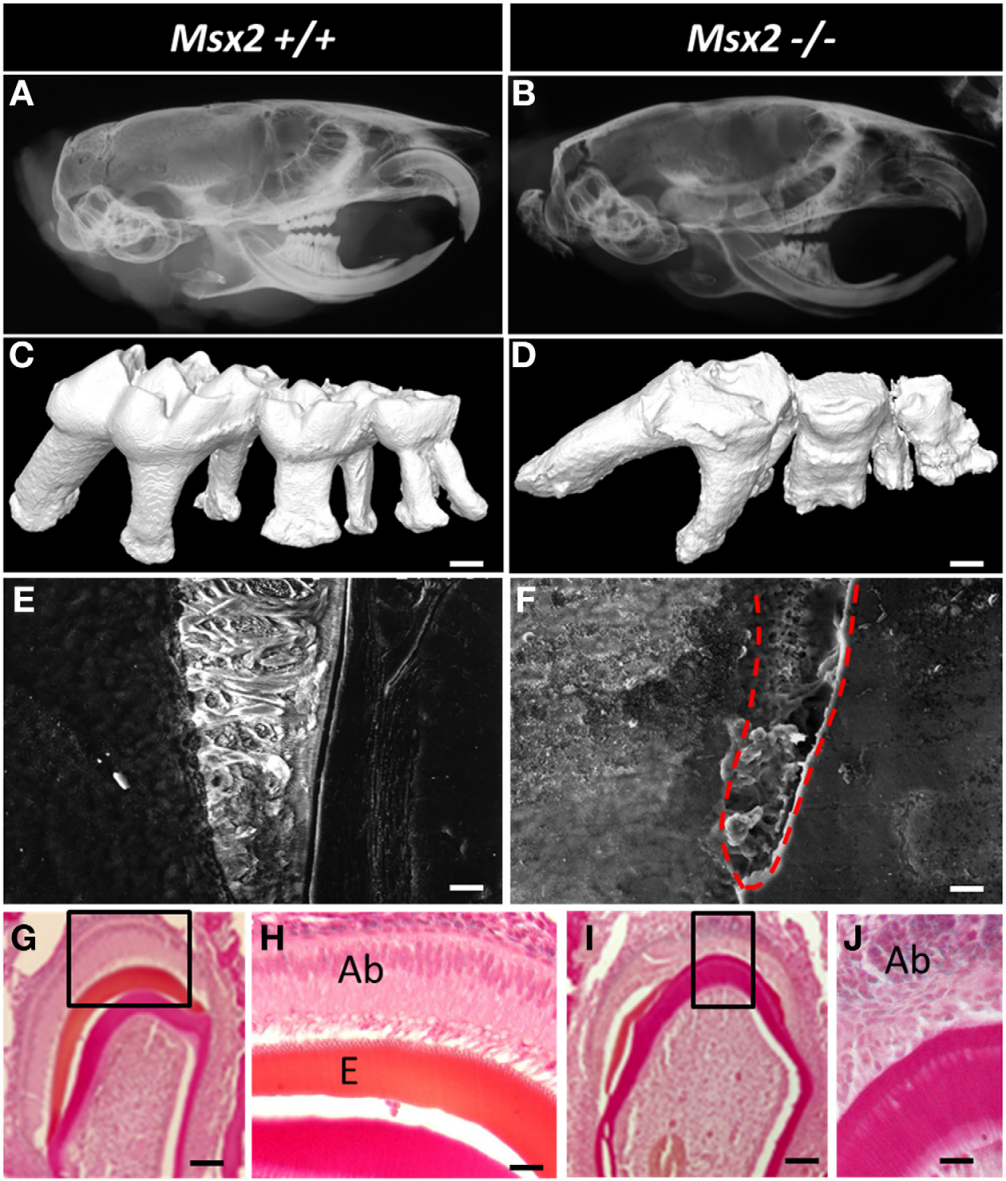
**Dental phenotype of *Msx2*−/− mice with reference to wild-type animals (*Msx2*+/+). (A,B)** Microradiographs of the whole heads of 3-month old mice showing craniofacial and teeth dysmorphology; indeed craniofacial morphogenesis is under the control of MSX2 (Simon et al., 2014). *Msx2*−/− mutant mice present a non-isometric overall craniofacial size decrease; the teeth exhibit crown and root dysmorphology with altered enamel, enlargement of the pulp cavity, short and curved roots with abnormal orientations, and reduced curvature of the incisor. The third molar showed impaired eruption and the most severe phenotype. **(C,D)** 3D reconstruction of mouse molars revealed the absence of cuspid relief and severe generalized enamel hypoplasia with irregular surface. *Msx2*−/− mice displayed complex radicular morphology (Aïoub et al., 2007). **(E,F)** Scanning electron microscopy of the first molar mandible illustrates a severe reduction in enamel thickness. Enamel in *Msx2*−/− animals shows the absence of enamel prisms, replaced by an amorphous layer (Molla et al., 2010); scale bars: 10 μm. **(G–J)** Histological analysis of mouse molar enamel reveals hypoplastic amelogenesis imperfecta in *Msx2*−/− mice. This feature is related, after a correct ameloblast differentiation process, to a secondary inability of ameloblasts to secrete the enamel matrix which would mineralize. Ameloblast cells in these animals lose their polarization, become rounded and isolated, and finally disappear (Ab, ameloblast; E, enamel —scale bars: **G**, **I**: 100 μm; **H**, **J**: 40 μm).

At this time, several targets have been identified in enamel within their physiological context. Dental morphogenesis in *Msx2*−/− mice is abnormal: cusp generation is affected by enamel knot disorganization (Figure 3D) (Bei et al., 2004); defects are observed in the enamel (Molla et al., 2010) and other dental tissues; and roots are malformed with the root epithelium overexpressing enamel proteins, especially amelogenins and ameloblastin. Also, jaw osteoclast activity is decreased locally around the dental follicle; eruption failure and pseudo-odontogenic tumor deviation of the tooth germs culminate in the third mandibular molars (Aïoub et al., 2007). This phenotype is coherent with the pattern of *Msx2* expression in many cells forming the tooth and periodontal bone. Indeed, its expression starts from the very beginning of odontogenesis, first in the ectoderm and mesoderm from the gastrulation stage, and subsequently in neural crest cells and oral epithelium (Bendall and Abate-Shen, 2000). During root formation, *Msx2* is again expressed in epithelial cells (Hertwig root sheath and later epithelial Malassez rests) as well as in dental and periodontal mesenchyme (Yamashiro et al., 2003; Molla et al., 2010). As alveolar bone and tooth development are linked, it is important to note that differentiating and differentiated osteoblasts express *Msx2* (Dodig et al., 1996; Mirzayans et al., 2012). Finally, peridental osteoclasts express *Msx2* and do so with regional gradients related to both dental crown and root growth (Aïoub et al., 2007) (Figure 3).

In summary, all cells associated with the complex formed by tooth and bone express the *Msx2* gene during their lifetime, and do so with exquisitely precise timing and levels. This makes it difficult to directly anticipate MSX2 function in one tissue (*in vivo*) from data obtained *in vitro*. For example, in the periodontal ligament cells, MSX2 prevents osteo-differentiation *in vitro* (Yoshizawa et al., 2004) while bona fide ankylosis is not found in *Msx2*−/− mice (Aïoub et al., 2007). A thorough analysis of MSX2 function in tooth/bone inter-dependent development requires a number of conditional gene mutations or misexpressions in each MSX2-target cell, each corresponding to different restricted temporal windows and finely defined levels of expression. A complementary strategy has been used to rescue osteoclast activity in non-conditional *Msx2*−/− mice by mating *Msx2*−/− mice with a transgenic mouse line overexpressing RANK (Tnfrsf11a), the main osteoclastic-differentiating factor (Castaneda et al., 2013). This allowed the impact of one-cell processes (resorption) on tooth and bone complex formation to be specified and described.

### *Msx2* controls morphogenesis via epithelial *Bmp4* levels and the associated death program in enamel knot

Early tooth development in *Msx2* null mice is normal, and only a small number of effectors are modified: epithelial *Bmp4* expression decreases whereas expressions of *Fgf4*, the cyclin-dependent kinase (cdk) inhibitor gene p21, and *Shh* are not modified (Bei et al., 2004). Furthermore, *Bmp4* expression is not altered in the mesenchymal compartment. MSX2 intervenes in epithelial-mesenchymal cross-talk, leading to odontogenesis. Mesenchymal BMP4 stimulates *Msx2* and *Cdk* p21 expression in the enamel knot; MSX2 in turn stimulates *Bmp4* expression in epithelial cells. MSX2 *in vitro* cooperates in the BMP4-mediated programmed cell death pathway (Israsena and Kessler, 2002), and *Msx2* overexpression stimulates *Bmp4* increasing apoptosis (Marazzi et al., 1997; Wu et al., 2003). Thus, the regulatory loop initiated by MSX2 is determinant for dental cell signaling and communication and consequently tooth morphogenesis.

### Altered laminin 5α3 patterns affect ameloblast integrity

**Figure 4:**
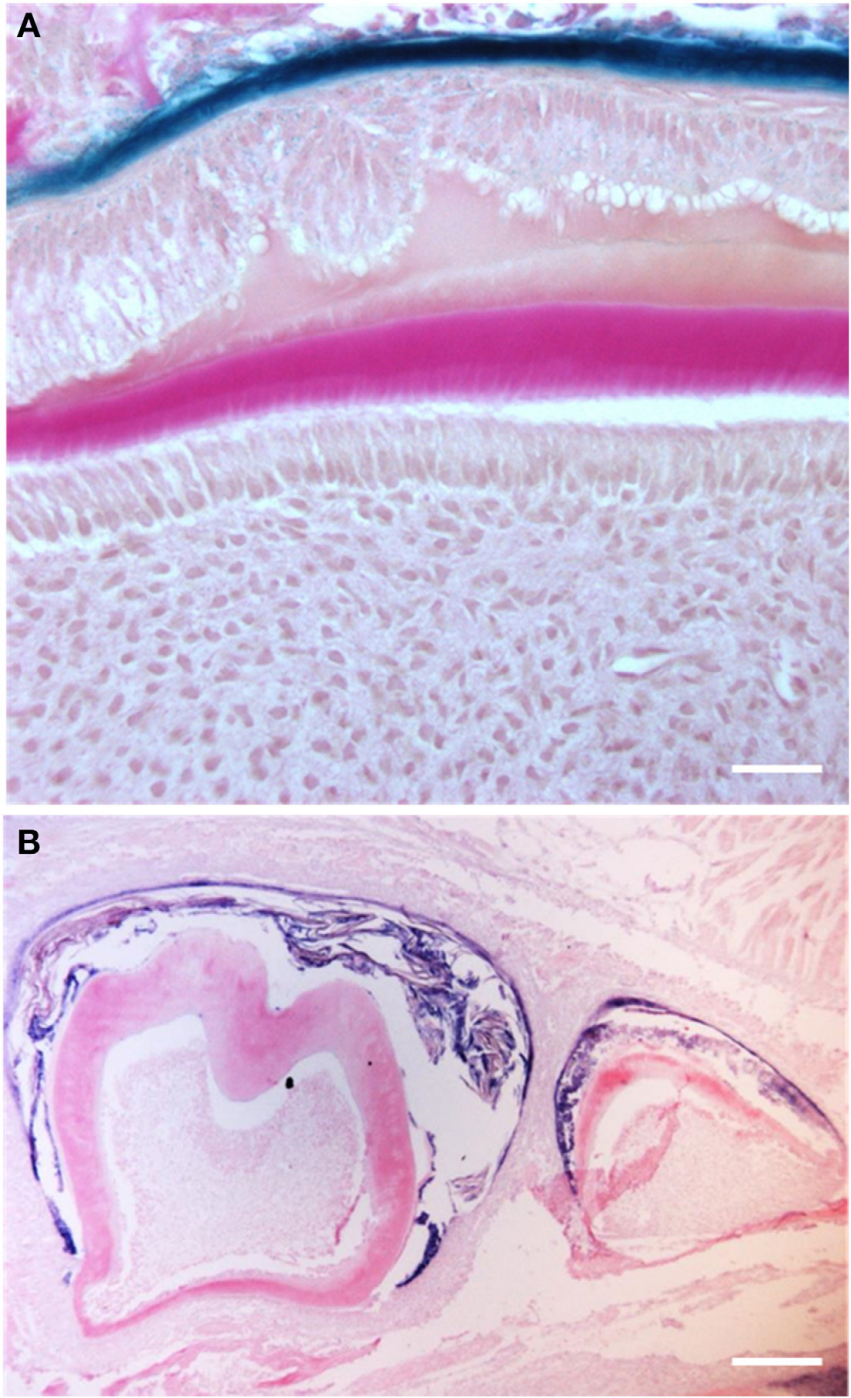
**Amelogenin production in 21-day old *Msx*2−/− mice showing ameloblast cell secretory disorders in more detail (Molla et al., 2010). In this condition, the polarity of the entire enamel organ is completely lost. (A)** Epithelial cells appear to delaminate losing their orientation and intercellular cohesion (scale bars: 20 μm). **(B)** The apparent level of amelogenin RNA synthesis is uniformly low in the entire enamel organ. The resulting ameloblasts do not produce significant amounts of enamel matrix (scale bars: 100 μm).

In the dental epithelium, laminin 5α3 is expressed in the basal membrane prior to ameloblast differentiation and disappears when cells differentiate (Yoshiba et al., 1998). It has been described as being distributed in a “membrane like” structure localized between the stratum intermedium and ameloblast cell layer. In *Msx2*−/− mice, ameloblasts are able to achieve terminal differentiation but the integrity of their junctional complexes is affected. In the absence of MSX2, the inner dental epithelium presents rounded and detached ameloblasts with loose intercellular junctions (Bei et al., 2004; Molla et al., 2010) (Figure 4). Laminin 5α3 expression is lower than in wild-type animals, whereas the expression of E-cadherin, β-catenin, and the integrin subunits α5β 5 and α6β 4 remains unchanged (Bei et al., 2004; Molla et al., 2010). Thus, the MSX2 target gene laminin 5α3 may control the formation of cell-cell-junctions and thus organization of the ameloblastic layer (Zhou et al., 1999). This possibility is supported by a *LAMA5* gene mutation in the epidermolysis bullosa characterized by skin fragility and enamel dysplasia (Brooks et al., 2008), as a result of the destruction of dermal and dental epithelia, respectively.

## Prospects for experimental models—from discrete to continuous parameters of enamel quality control

### The physiological conditions are only partly reproduced *in vitro*

There have been reported successful *in vitro* promoter studies in ameloblasts (see above) thanks to establishment of cell models that help to decipher molecular mechanisms (Zhou and Snead, 2000; Zhou et al., 2000; Xu et al., 2007a). However, the transcriptional program leading to ameloblast activity during enamel presecretion, secretion, and post-secretion is only progressively emerging (Lacruz et al., 2012b; Simmer et al., 2014) (Figure 2). Indeed, the spatial and temporal program of enamel formation is not recapitulated in cell cultures. Five factors are more or less reproduced in current cell culture models: (1) Epithelial organization and ameloblast polarity. (2) Signals produced by non ameloblastic cells (enamel knot, stratum intermedium and mesenchymal odontoblasts) which drive ameloblast fate and activity. Indeed, epithelial-mesenchymal interaction has been demonstrated in ameloblasts co-cultured with odontoblasts (Matsumoto et al., 2011). (3) Key stages of presecretion, secretion and post-secretion related to up- and down-regulation of matrix protein levels and protease activities (Figure 2). (4) The delicate crystal and prism architecture which requires significant and ordered enamel matrix deposition and biomineralization. (5) An appropriate peridental microenvironment in which bone apposition and resorption rates may influence enamel formation. Indeed, allometric tooth growth is dependent on (and reversely determines) eruption rate (Castaneda et al., 2011, 2013).

### The rodent incisor, an “*in vivo* test tube” for analyzing gene and environment interactions in enamel

Various experimental approaches have been developed including organotypic cultures (Bronckers, 1983; Bronckers et al., 2009), primary cultures (DenBesten et al., 2005), and hybrid cell cultures (Matsumoto et al., 2011; Jiang et al., 2014). *In vivo* studies may involve dissections under a microscope which is either manual or, more recently, by laser-capture (Jacques et al., 2014). For growth-limited teeth (three non-renewed molars in rodents), amelogenesis follow-up requires animals of different ages, increasing the sample size required (Onishi et al., 2008). Rodent incisor enamel displays an exceptionally simple structure and is reasonably large. Its continuous growth facilitates the exploration of enamel formation under defined conditions and during defined temporal windows (transgenic mice, environmental, and systemic disturbances) at any animal age (Damkier et al., 2014). The course of amelogenesis is spatially distributed along the longitudinal axis of the tooth. Consequently, extracellular cascades of peptide-peptide and mineral interactions can be sampled along the longitudinal axis of single rodent incisors (Jedeon et al., 2013), and ameloblasts and changing enamel matrix are easily followed (Houari et al., 2014). Protein and mRNA studies are feasible and have included comparisons between incisor samples from test and control conditions in rats (Berdal et al., 1993; Jedeon et al., 2013) and mice (Descroix et al., 2010; Molla et al., 2010).

### MSX2 is a morphogen for enamel, controlling its thickness

**Figure 5:**
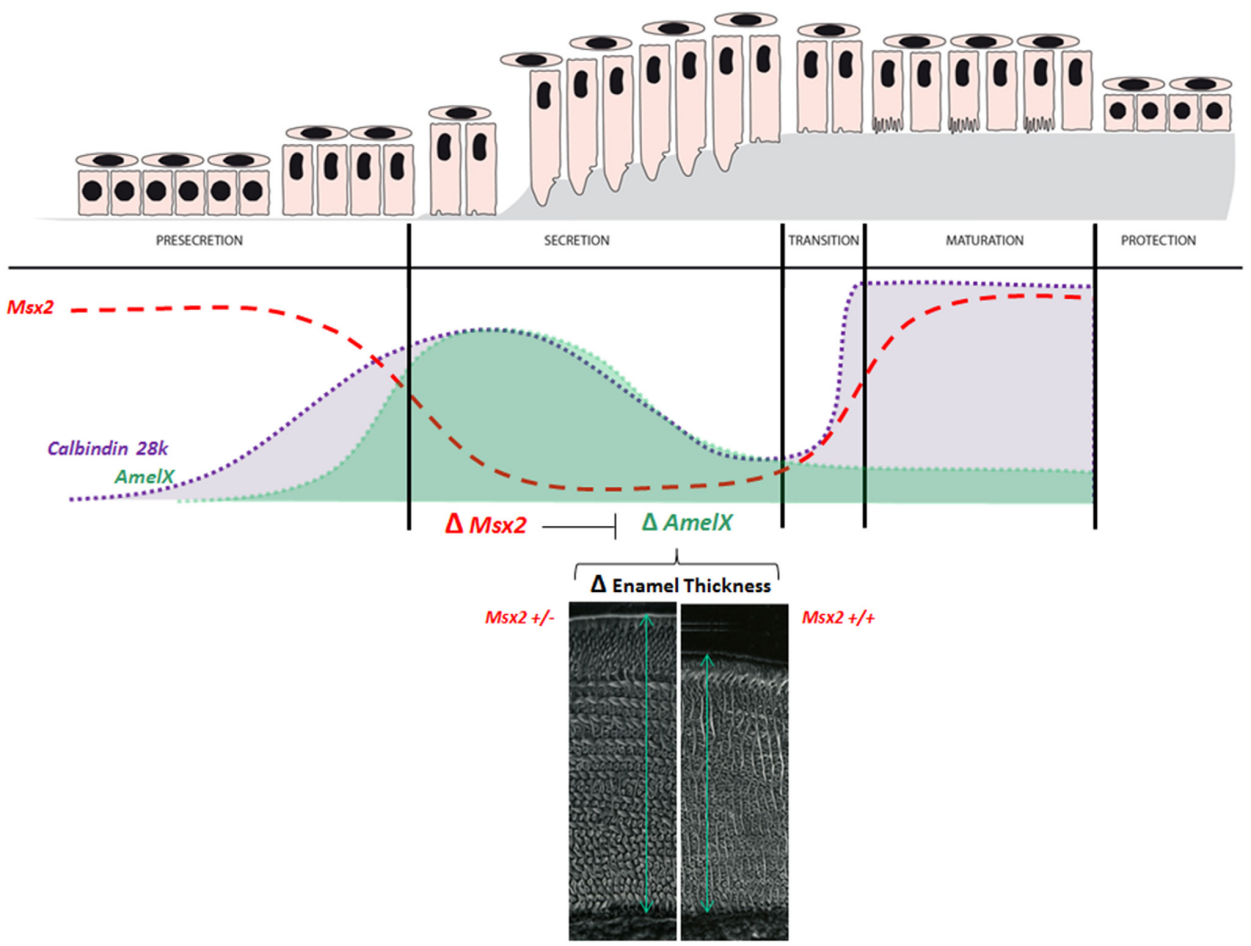
**Physiological levels of MSX2 and its target-genes, amelogenin and calbindin-D_28k_**. *Msx2* expression during the ameloblast life-cycle is inversely related to amelogenin abundance. The figure is a compilation of published findings (Molla et al., 2010) and illustrates two significant examples of gene expression level fluctuations (amelogenin—green; and calbindin-D_28k_—gray) in ameloblasts during amelogenesis and their relationships with *Msx2* expression (red). (1) The presecretion/secretion/maturation sequence. *Msx2* down-regulation is related to down-regulation of amelogenin and calbindin-D_28k_ from the presecretion stage to the secretion stage. (2) During the secretion stage controlling enamel thickness; amelogenin mRNA production in ameloblasts depends on their sites or localization (Snead et al., 1988), leading to the differing thickness of enamel across the tooth. The patterns of *Msx2* and *Dlx2* expression are the converse of that of amelogenin (Lézot et al., 2008). We established that MSX2 indeed contributes to enamel thickness inhibition *in vivo* (Molla et al., 2010). Enamel thickness as determined by scanning electron microscopy in mandible incisor of 3-month old mice is greater under *Msx2* haploinsufficiency than in controls (here *Msx2*+/− compared to wild-type *Msx2*+/+ mice). This Msx2 haploinsufficiency is also associated with overexpression of both amelogenin (Molla et al., 2010) and calbindin-D_28k_ (Bolaños et al., 2012). The relationship between calbindin-D_28k_ and MSX2 is more complex because calbindin-D_28k_ abundance abruptly increases for a second time during the maturation stage. However, the partners of MSX2 at this stage are still unknown and its activity on gene expression has not been extensively investigated.

The murine incisor “*in vivo* test tube” has been used in studies of MSX2 in differentiated ameloblasts. *Msx2* heterozygous (*Msx2*+/−) mice are a unique model for investigating MSX2 dose effects. Ameloblasts survive and differentiate appropriately in *Msx2*+/− mice (unlike those in *Msx2*−/− mice) and the amounts of *Msx2* mRNA are half those in wild-type mice. Enamel gene studies have revealed a specific overabundance of amelogenin mRNA in *Msx2*+/− mice. The enamel phenotype in *Msx2*+/− mice included increased thickness and, more specifically, a thicker outer prismatic enamel layer and larger prism diameter (Molla et al., 2010; Figure 5). This suggests that MSX2 determines enamel thickness *in vivo*. Indeed, a rigorous analysis of the pattern of *Msx2* expression during the secretion stage revealed a inverse relationship between *Msx2* mRNA levels and enamel thickness (Molla et al., 2010) as similarly described for enamel thickness and amelogenin levels (Snead et al., 1988). This is also the case for DLX2 (Lézot et al., 2008) in which an inverse correlation between DLX2 levels and thickness was shown through quantitative measurements of incisors. Also, decreased production of enamel proteins during the enamel maturation stage is associated with a significant up-regulation of *Msx2* (Figure 5), in accordance with *in vitro* data showing the MSX2 transcriptional repression of amelogenin expression. On the contrary, ameloblast alterations observed in *Msx2*−/− mice result in deficient enamel protein production (especially amelogenin) and result in hypoplastic enamel without visible prismatic structures (Molla et al., 2010—see Figure 3F).

## Conclusion

Integrative physiological networks underlying amelogenesis are just emerging. Experimental progress in the field of enamel has mainly been based on gene disrupting strategies to describe developmental circuits which drive enamel formation. However, only precise quantitative and continuous studies allow appropriate analysis of the interplays that determine enamel thickness and quality. This is the case for studies addressing the constitutive regulation of ameloblast activity, illustrated here by the MSX and DLX homeoprotein pathways. They provide clues on how enamel acquires differential thickness depending on its anatomical location. They modulate expression of key genes involved in amelogenesis (Lézot et al., 2002, 2008; Molla et al., 2010; Bolaños et al., 2012). It is thus very likely that these factors are able to transmit effects of many environmental factors, whether regional or systemic (including the availability of calcium, vitamin D, fluoride, or even pollutants) that also affect the final enamel composition and quality (Berdal et al., 1993; Jedeon et al., 2013; Houari et al., 2014).

In summary, the dental cell and enamel system illustrates how MSX homeoproteins may be reiteratively enrolled in a single organ. Throughout the cell life-cycle, cooperation between particular transcription factors in a stage-specific manner controls the expression of a number of genes. Such MSX2-target genes encode growth factors, junctional complexes, matrix proteins, and other proteins involved in enamel mineralization. Consistent with reiterative up- and down-regulations, MSX2 drives particular events; some in early development, some during growth and, finally, some in conditions of homeostasis in adults with the effect decreasing with age. MSX2 is exemplary of the integrative schemes of a single transcription factor making multiple contributions to the physiological or pathological development of complex organs, composed of many cells of different types, as described here in the dental-bone complex.

## Funding

This work was funded by the University Paris-Diderot, the French National Institute of Health and Medical Research (INSERM) and the National Research Agency (ANR): OSTEODIVERSITY (SVSE 1-2012).

### Conflict of interest statement

The authors declare that the research was conducted in the absence of any commercial or financial relationships that could be construed as a potential conflict of interest.

## Abbreviations

Bp: Base pairs
IEE: internal enamel epithelium.
